# Perceived Risk of Mosquito-Borne Arboviruses in the Continental United States

**DOI:** 10.3390/pathogens10121562

**Published:** 2021-11-30

**Authors:** Saul Lozano, Jonathan Day, Lilyana Ortega, Maggie Silver, Roxanne Connelly

**Affiliations:** 1 Division of Vector-Borne Diseases, Centers for Disease Control and Prevention, Fort Collins, CO 80521, USA; uvt8@cdc.gov (M.S.); csz5@cdc.gov (R.C.); 2 Florida Medical Entomology Laboratory, University of Florida, Vero Beach, FL 32962, USA; jfda@ufl.edu; 3 College of Health and Human Sciences, Colorado State University, Fort Collins, CO 80523, USA; lilyana.ortega@colostate.edu

**Keywords:** risk perception, vector-borne diseases, West Nile Virus, Zika Virus

## Abstract

The United States experienced local transmission of West Nile Virus (WNV) for the first time in 1999, and Zika Virus (ZIKV) in 2016. These introductions captured the public’s attention in varying degrees. The research presented here analyzes the disproportional perception of ZIKV risk compared to WNV transmission risk, by the public and vector control personnel. The risk perception of vector control was measured through purposive sampled interviews (24 interviews in 13 states; May 2020–June 2021), while the public’s perception was estimated from news publications (January 2000–December 2020), and Google searches (January 2004–December 2020). Over time, we observed a decrease in the frequency of press reporting and Google searches of both viruses with decreasing annual peaks in the summer. The highest peak of ZIKV news, and searches, surpassed that of WNV. We observed clear differences in the contents of the headlines for both viruses. We propose that the main reason in risk perception differences between the viruses were psychological. Zika infections (mosquito-borne and sexually transmitted) can result in devastating symptoms in fetuses and newborns, observations that frequently appeared in ZIKV-related headlines. Our results highlight the likely influence the news media has on risk perception and the need for public health agencies to play active roles in the conversation, helping disseminate timely and accurate information. Understanding the factors that shape risk perceptions of vector-borne diseases will hopefully lead to better use of resources by providing better guidance.

## 1. Introduction

More than five-hundred years ago yellow fever and dengue arboviruses reached the Americas from Africa. It was not until the 20th and 21st centuries that the United States experienced the introduction of several more arboviruses. West Nile Virus (WNV) was first detected in New York City in 1999. An average of 28 cases of travel-related Chikungunya virus (CHIKV) per year were detected in the United States between 2006 and 2016. Twelve locally transmitted cases of CHIKV were reported in Florida during 2014 [1]. Local transmission of Zika Virus (ZIKV) in the USA was first detected in 2016 in South Florida. These recent arboviral introductions have captured the public’s attention; however, public knowledge about the viruses has not translated into support for vector control activities or the adoption of protective “health behaviors” [2]. A health behavior refers to any behavior that influences, or is believed to influence, physical health outcomes, either by increasing or decreasing an individual’s disease risk or severity of disease (e.g., using mosquito repellent).

Risk perception, including how susceptible or serious a disease might be, is often featured in behavioral models and theories to predict, explain, or change health behaviors. Understanding how the public perceives risk can be an important step to change health behaviors. However, risk perception is influenced by many factors. For example, uncontrollable or involuntary threats tend to cause more anxiety among the public than threats that can be controlled or responded to voluntarily [3]. Not only does the novelty and degree of uncertainty about a threat influence the perception of risk [4,5], but so does the individual’s worldview and their trust in the recommendations of experts or management authorities [6]. Understanding risk perception can aid in presenting data and health behavior change strategies in more effective ways.

In this manuscript we assess the risk perception towards WNV and ZIKV by vector control agencies and the public, evidencing that WNV and ZIKV elicited different responses.

## 2. Results

### 2.1. Distribution of West Nile and Zika Cases in CONUS

After an explosive expansion between 1999 and 2004, WNV has become endemic throughout most of the CONUS (Table 1; data source: ArboNET). Using only the most severe cases of WNV by county, we observed that neuro-invasive cases were recorded for the first time in a large number of counties and states in CONUS between 1999–2004 (Table 1, gray column). The most recent reports of neuro-invasive WNV were similarly wide-spread across the CONUS (Table 1, white column); a large portion of the CONUS has been continuously affected by WNV since its introduction in 1999.

Zika Virus was first detected in CONUS in 2016 (Table 2, gray columns; source: ArboNET). In addition to reports of locally acquired mosquito-borne transmission in Florida and Texas, ZIKV was sexually transmitted, and had devastating consequences on developing fetuses. Locally acquired cases (i.e., cases with no travel history) included mosquito-transmitted infections and sexually transmitted infections. States with locally acquired mosquito transmitted cases (*n* = 231) are identified as “*” in Table 2; cases were restricted to Florida and Texas. Sexually transmitted cases (*n* = 47) were widely dispersed (22 states); these cases are considered sexually acquired because the patient had no travel history and no known exposure to mosquito vectors. By comparing Table 1 and Table 2 it is clear WNV is a frequent and widespread threat within the United States (at the selected scales), while ZIKV only appeared sporadically.

### 2.2. Vector Control Agencies Perception of Risk

**Figure 1:**
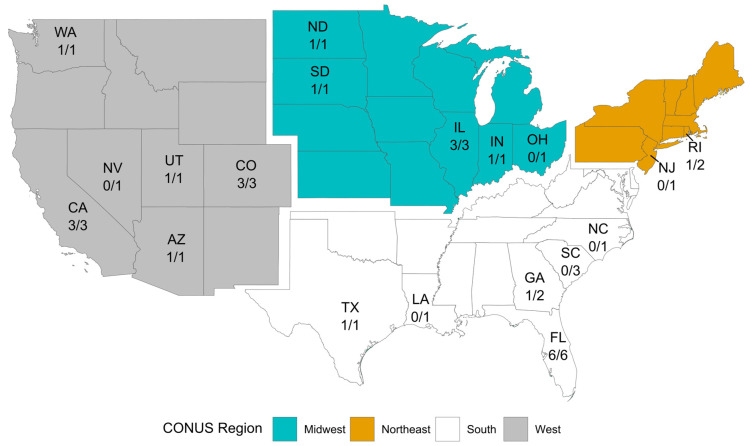
Location of contacted and surveyed vector control agencies by state (ISO 3166-2). The numerator denotes the agencies interviewed by the time of manuscript preparation while the denominator is the total number of agencies contacted.

A total of 24 interviews (out of 34 contacted) were conducted throughout CONUS (Figure 1) between May 2020 and June 2021 to evaluate how vector control agencies and personnel perceive the risk of WNV and other vector-borne diseases within their jurisdictions. Twenty-three of the twenty-four agencies were in counties with at least one neurological WNV case between 2014 and 2019. In contrast, only a few of the interviewed agencies had local mosquito or sexually transmitted Zika cases. As previously mentioned, cases acquired through sex were widely dispersed while mosquito-transmitted cases were limited to Florida and Texas.

**Figure 2:**
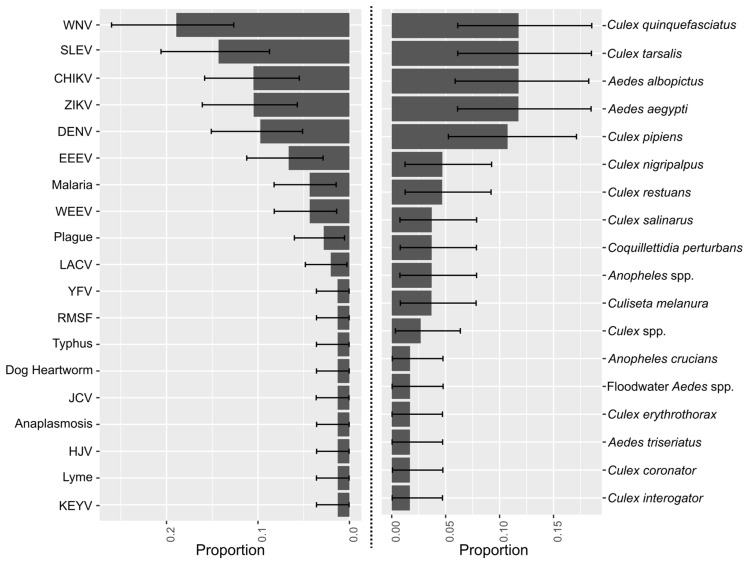
Multinomial analysis of vector-borne diseases of concern and the vectors species of concern within their jurisdiction. Error bars represent 95% credibility intervals. WNV = West Nile virus; SLEV= St. Louis encephalitis virus; CHIKV = Chikungunya virus; ZIKV= Zika Virus; DENV = dengue virus; EEEV = eastern equine encephalitis virus; WEEV = western equine encephalitis virus; LACV = La Crosse virus; YFV = yellow fever virus; RMSF = Rocky Mountain spotted fever; JCV= Jamestown Canyon virus; HJC = Highland J virus; KEYV = Keystone virus.

Among all the interviewees, the viruses (used interchangeably with vector-borne diseases) most frequently mentioned (and of greatest concern) were WNV followed by St. Louis encephalitis virus (SLEV; Figure 2). However, no statistical differences were detected (i.e., uncertainty bars overlapped) between WNV, and SLEV, ZIKV, dengue viruses (DENV), CHIKV, or eastern equine encephalitis virus (EEEV) as viruses of concern.

Corresponding with the WNV and SLEV mentions, the vector species of most concern were *Culex quinquefasciatus* Say and *C. tarsalis* Coquillett (Diptera: Culicidae). However, *Aedes aegypti* L. and *A. albopictus* Skuse (Diptera: Culicidae) had the same proportion of mentions to these *Cx*. species. *Culex quinquefasciatus* mention was statistically similar to most species with the exception of *C. erythrothorax* Dyar, *C. coronator*, *C. interrogator* Dyar and Knab (Diptera: Culicidae), and *Anopheles crucians* Wiedemann (Diptera: Culicidae). To summarize, the agencies’ concern for DENV and ZIKV vectors was statistically similar to that for WNV vectors. Furthermore, WNV was statistically similar to DENV and ZIKV in terms of worry.

### 2.3. Public Perception of Risk (Headline and Search Term Analysis)

A total of 43,265 articles were found for WNV and 15,495 for ZIKV in the “USNews” database. However, after data scrubbing (amending or removing headlines that were incorrect, incomplete, improperly formatted or duplicated; see Figure S1) the total was reduced to 37,239 and 12,977 for WNV and ZIKV, respectively. We obtained headlines from 362 uniquely identified newspapers (complete list Table S1); the newspapers that contribute the most headlines (top 15) are presented in Table 3 for WNV and Table 4 for ZIKV. The newspaper that provided the most WNV headlines was the *Daily Herald* (Chicago, IL, USA), closely followed by the *Newsday* (New York City metro area, NY, USA), and the *Boston Globe* (Boston, MA, USA), the *Chicago Tribune*, and the *New York Times*. However, the three top newspapers account for only 8% of the WNV headlines and the top 15 newspapers accounted for only 28% of the headlines. In contrast to the WNV headlines, the three newspapers that provided the most ZIKV headlines were the *Wall Street Journal*, the *New York Times*, and *The Washington Post*. These three newspapers are in the top-ten most circulated newspaper in the United States (U.S.), and provided 13% of the ZIKV headlines. The USA Today, with the largest newspaper circulation in the US, did not appear among the top 15 in the WNV dataset, but was 8th in the ZIKV dataset.

**Figure 3:**
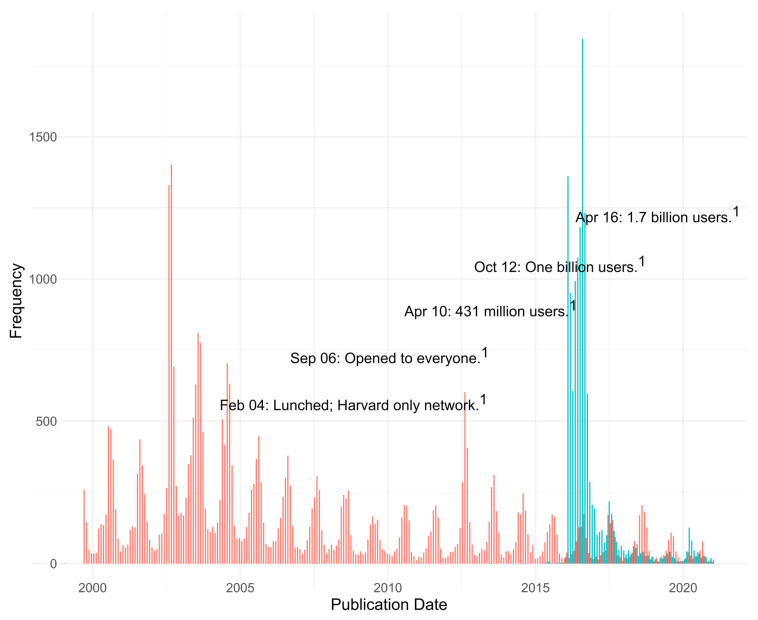
Monthly frequency of articles published with ●WNV or ●ZIKV in their full text or headline contrasted with ^1^ Facebook milestones [16].

The first mention of WNV in our dataset was on 25 September 1999 when we identified six WNV-related news articles. Among them we identified one article that reported the deaths of three people in New York City that may have been caused by WNV. This was the first report of WNV transmission in the Western hemisphere [7]. By the end of 1999, it was clear that these and other reported deaths were the result of WNV infection [8]. On its initial entry into the CONUS (1999), WNV received press coverage (1999; Figure 3 red bars), but the coverage was more extensive in 2000 and 2001. The most extensive coverage of WNV in the press was reported in August of 2002 when WNV was detected in 40 states [9]. After 2002, the frequency of WNV press reports followed a similar pattern with annual peaks in August or September. There was a decline in the number of WNV-related news articles in 2009 and 2010. In 2012 we observe a peak of WNV press reports corresponding to an epidemic in Texas [9]. The 2012 reports were equivalent to the number of WNV press reports in 1999. After 2012, there was a decrease in the number of WNV newspaper articles with the lowest number of reports recorded in 2020.

The first mention of ZIKV in the USNews database was in June 2007 in the Pacific Daily News published in Hagatna, Guam [10]. The article describes “An outbreak of illness on the island of Yap”. A few days later a NYT article describes the ongoing outbreak in Yap [11]. The Pacific Daily News published three more articles about ZIKV in 2007. It was not until 2013 that an article in the World Watch section (a news compilation of world news) of the *Chicago Tribune* next mentioned ZIKV. The article, originally published in Thailand, reported that a Canadian tourist returning from Bangkok and Phuket was diagnosed with ZIKV [12]. In 2014 the *Chicago Tribune* described a ZIKV outbreak in New Caledonia (140 cases) using case numbers reported by the CDC. Most of the New Caledonia cases were locally acquired, but some were contracted in French Polynesia [13]. In 2015 a total of 33 articles where found, however most of them centered on the question of why some people are attractive to mosquitoes. In July 2015, the *Chicago Tribune*’s World Watch section reported a large outbreak of ZIKV in the Pacific Islands. A second article, completely dedicated to ZIKV, appeared in *The Washington Post* (online) in September 2015 [14]. The article mentioned that ongoing transmission of ZIKV was reported in South America and Oceania, and that ZIKV was the first known mosquito-borne virus that could also be sexually transmitted [14]. The first article that mentioned a link between a “dengue-like virus to birth defects in babies” in Brazil was published in November 2015 [15]. By 2016 the number of ZIKV articles jump to 10,491. Seventy-four percent of the ZIKV articles published so far appeared in 2015 (Figure 3, blue bars). The peak of ZIKV reports was in August 2016 (1881 articles), surpassing all previous monthly WNV press releases. Early in 2016, many large circulation newspapers reported on the association between microcephaly and ZIKV (and the mosquito transmission of ZIKV). After, 2016, the overall number of ZIKV publications decreased. Summer peaks of ZIKV reporting appeared in August 2017–2020. In 2020, there was an increase in ZIKV press reports compared to 2019. However, most of the 2020 articles referenced ZIKV as an example of a recent pandemic.

**Figure 4:**
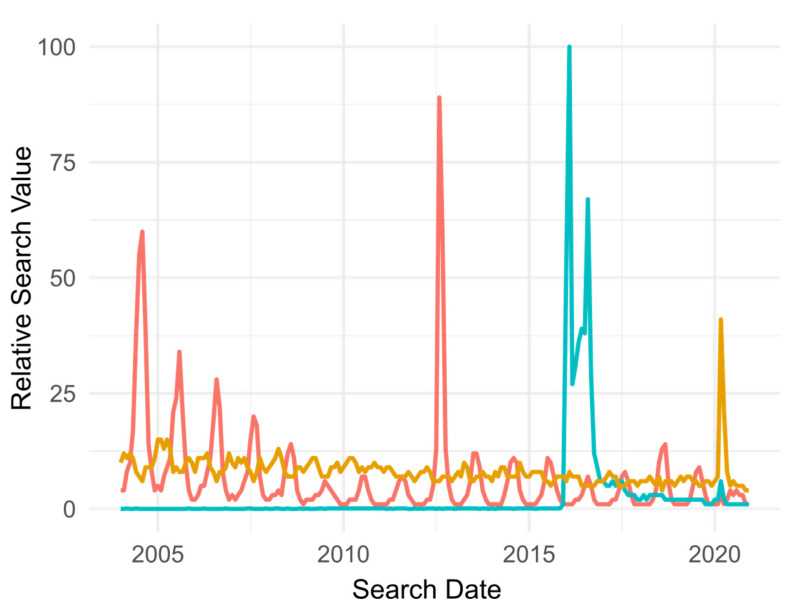
Monthly number of searches for the topics, ● “West Nile”, ● “Zika”, and ● “Malaria”. The value is relative to the month with the largest number of searches.

In addition to looking at news article mentions, we also obtained from Google Trends the relative search values (calculated by comparing between monthly search totals, the month with the most searches is given a 100% value,) for WNV, ZIKV, and malaria (as a normalizing variable). Figure 4 shows the monthly relative search value for West Nile Virus (red), Zika Virus (blue), and malaria (yellow) from 2004 through 2020. The highest peak for ZIKV was in February 2016 (100%). The second largest peak was for WNV in August 2012 with a value of 89%. The malaria search peak observed in 202 is likely due to articles mentioning an antimalaria medication as a treatment for COVID-19. Interestingly, the patterns observed in the headline analysis matched the patterns observed in the searches performed at Google for the years covered in our study. Public searches for WNV and ZIKV peaked in August or September every year, with a striking similarity to the frequency of newspaper articles, suggesting that the relative interest in ZIKV was higher than that for WNV. A small difference between the number of published press releases and the number of Google searches was observed in 2012, when the WNV searches were higher than the searches in 2004, while the number of published articles in 2012 was lower than the number published in 2004 and 2005.

### 2.4. Word Cloud Analysis of the Headlines

**Figure 5:**
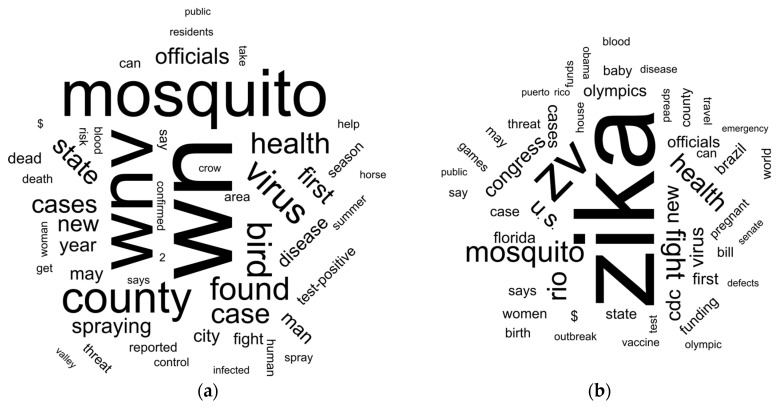
Word clouds for the 50 most frequent terms found in newspaper articles headlines: (**a**) WNV headlines; (**b**) ZIKV headlines.

We created word clouds with the fifty most common terms found in the WNV and ZIKV headlines to assess the headline contents (Figure 5). Though we present the disease terms (e.g., West Nile = wn) and the virus terms (e.g., WNV = wnv) separately, they were used interchangeably in the headlines. The term “virus” was commonly used in the headlines to avoid repeating the disease in publications with two-part titles; “virus” was also commonly used in place of WNV or ZIKV (e.g., “New Virus in Town”, “Little-known Virus”, or “Dengue-like Virus”).

The most frequently used term in WNV headlines was “wn” closely followed by “mosquito”, apparently showing an association between the virus and mosquitoes. The term “bird” (and “crow”) was also found with a relative high frequency. In contrast, ZIKV headlines most commonly used the term “zika”, with considerably fewer mentions of “mosquito” as compared to WNV headlines. The ZIKV word cloud analysis also included the terms “pregnant”, “baby”, and “birth defect”.

Another interesting difference between the two disease word clouds is the mention of governmental organizations. The WNV headlines mentioned “county” and “state” prominently, while the ZIKV headlines mentioned congress (“congress”, “house”, and “senate”) frequently. The term “obama” (*n* = 136) was one of the most common terms in the ZIKV cloud. However, there was not a unique topic associated with the term “obama”. Some of the headlines contain “Obama administration …”, or “Obama’s budget …”, or “Obama to take (aggressive, robust, etc.) action ..”. The article “Obama Raps Congress Over Zika Funding” was published 34 times. The term “obama care” was not found while “obamacare” was found only five times.

Another clear difference between the two clouds is the mentions of the Rio de Janeiro Olympic games in the ZIKV cloud. The ZIKV pandemic coincided with the Olympic games, and Rio was considered a future epicenter of the virus. Other specific places mentioned in the ZIKV cloud were Puerto Rico, U.S., Florida, and Brazil, while no specific place was observed in the WNV cloud. Among the curious findings in the headlines is that “$” was commonly found in both clouds. The number “2” was frequent in the WNV headlines (e.g., “2 more cases in …”), but it was not as prevalent as the term “first” announcing a “first case” in humans or horses.

As expected, by their nature, headlines did not provide much information. For example, trying to extract the knowledge that WNV can be found in mosquitoes, we marked the headlines that contained the verb “found” (*n* = 1644); prominently display in the WNV cloud. The term that commonly followed “found” was “in”. We found 1308 headlines that had “found in”. However, “found in mosquito[es]…” was only part of 100 headlines, which is low considering the total number of headlines analyzed (*n* = 37,239).

## 3. Discussion

The research presented here originated from observing a disproportional perception of risk to ZIKV, compared to the more prevalent WNV, in the interviews of the vector control agencies. We speculated that the perception could be related with, and maybe inflated, by the news. Our analysis is a first approach to the complex issue of risk perception. The risk perception of vector control agencies was measured by directly interviewing personnel at vector control programs across the country. To measure the public’s perception, we calculated the frequency of news articles publications, did a headline analysis and queried Google trends to discover the frequency of searches for WNV and ZIKV.

Vector control agencies in CONUS considered CHIKV, DENV, or ZIKV to be of concern like WNV even though, apart from a few areas in the southern United States, the chance of local transmission of CHIKV, DENV, or ZIKV at the time of the surveys was very low; this was a surprising finding. This concern, we could argue, might be a reflection of the unclear vectorial capacity of *A. albopictus* in regions of the United States where it is abundant. However, vectorial capacity would not explain the number of *A. aegypti* mentions. We could venture that the number of mentions were due to the invasive nature and history of the species in the United States. The perceived risk by vector control personnel of *Aedes aegypti*, and the diseases it could vector in CONUS, would be understandable as a long-term concern.

How humans react to risk depends on how the risk is perceived, regardless of the actual level of risk [3,17,18,19]. In general, humans tend to over-react to intentional actions and tend to under-react to natural phenomena and accidents [20]. For example, consider the public’s reaction to naturally occurring anthrax cases vs. cases caused by its deliberate release. The transmission of vector-borne diseases is clearly a natural phenomenon. In this regard, we would expect no difference in risk perception between WNV and ZIKV; both should be considered by the majority of the public as a natural phenomenon. It is important to understand the association between naturalness and risk because the perception of vector-borne disease transmission being a part of nature can lead to complacency [21].

The decline over time in the public’s interest in WNV and, to a similar extent, ZIKV may be explained by the tendency of people to over-react to immediate or newly identified threats and under-react to long-term threats [17,22]. We believe one reason WNV is currently not being perceived as an immediate threat is because it is established in the U.S., regardless of the cyclical nature of the disease. Since the introductions of WNV and ZIKV, the number of press releases and searches has decreased (except for the summer 2012 and the summer 2020 for WNV and ZIKV, respectively) with oscillations that peak during the summer months. Articles and searches of ZIKV in 2020 increased because it was used as an example of a recent public health threat.

One advantage concerning the promotion of mosquito control and community acceptance is that the human brain tends to over-react to changes that occur quickly [20]. The human brain is geared to detect fast changes compared to slow changes. This may favor mosquito control’s efforts to promote the use of protective measures and may explain the frequent use of the terms “first”, “2”, and “new” to announce the start of WNV outbreaks.

There are two human behaviors that could explain ZIKV’s increased risk perception. Risk is considered higher if there is the belief that it will impact future generations [20]. Additionally, we tend to perceive things that “offend our morals” (morality as is colloquially used) as having a high risk [17,23]. Events connected to children are particularly charged [24,25]. We propose that these human tendencies may be the main reasons that there was a higher perceived risk associated ZIKV transmission. This could also explain why in 2021 ZIKV is still considered a virus of public health concern by personnel at some vector control agencies. Another possible contributing factor to the higher perceived risk of ZIKV transmission was the disproportionate reporting of ZIKV and being easy to remember. This may have led to an overestimation of the risk of ZIKV transmission in the CONUS [26].

Headlines are one of the first things a reader sees when opening a news page. A headline can tell the reader the kind of article, and the tone for what is to follow. Headlines should also engage a reader; a person is more likely to continue reading if the headline provokes a reaction [27,28,29]. It is no surprise that terms that could provoke an overreaction are found in the word clouds. Terms such as “new”, “first”, “threat”, “risk”, “death”, “blood”, and “emergency” provoke a sense of immediate threat to humans and their environment. Terms that could elicit a moral response and threat to future generations such as “death”, “pregnant”, “baby”, and “birth defect” were also frequently found in the headlines. The presence of management authorities in the two clouds could be the result of the public’s interest in who was managing the disease emergency [6]. It would be inaccurate to claim that local or state governments were not engaged with the Zika risk response. We could speculate that the lack of vector control resources to manage a newly imported mosquito-borne disease—potentially vectored by container-breeding mosquitoes—overwhelmed local and state authorities and required a federal response. Hence, the large occurrence of terms related to the federal government in the ZIKV cloud.

Risk perception theory considers an individual’s beliefs about a threat and their beliefs about the authorities providing data, recommendations, and behavioral guidelines. As such, individual differences in beliefs will greatly impact risk perception and resulting behaviors. For example, if an individual does not trust government at the federal level [30], they are less likely to implement any behavioral recommendations provided by this entity [6]. However, the news plays a significant role in the awareness to health threats and the subsequent perception of risk. Additionally, while federal data, recommendations, and guidelines are valuable, it is imperative that public health agencies (at all levels) leverage the news, and search results, to disseminate accurate information. There should be a close relationship between public health organizations and the news media given the media’s control of the public’s attention, and the risk of misdirected or inaccurate messages leading to a distorted sense of risk. Regarding the impact of the messaging, communication at the local level is expected to have a greater impact because there is generally a higher level of trust in local authorities [2,31,32].

Our research should be considered with the following caveats. As with most survey derived data, our data should not be considered random, since the data resulted from agencies that were available (i.e., replied to survey requests). However, to improve the sampling we conducted “Purposive Sampling” [33] trying to cover several regions of CONUS. Since no previous risk perception data exist our survey is very valuable, akin to the first baseline survey of vector control agencies core competencies conducted in 2017 [34], but at a smaller scale. It is also important to consider that mosquito control agencies do not exist in a vacuum. They are subject to the opinion and concerns of the public [35] they serve, in addition to being exposed to the same media-generated information sphere as the public. Discerning which factor(s) influenced risk perception would be an important question to answer. Another caveat to be considered is that headlines provide limited information (when compared to the full article) and are geared towards catching the reader’s interest. Therefore, it is important to keep the reader engaged by provoking a reaction that could exaggerate the perception of the risk [26]. Another factor to consider is that and access to the internet and searching technology was not equally available to the public; access has dramatically increased between the introduction of WNV to CONUS and the ZIKV pandemic specially among the young [36]. Finally, it is possible that news articles may promote Google searches about specific topics. This situation could lead to news article and Google searches being correlated and not independent measurements of the public’s interest. However, because a search represents an individual’s action, searches offer a more direct link to a person’s interest, and therefore, we think, they should be considered when assessing the public health concerns of an area.

We believe the perception of risk by the public and by vector control agencies (in addition to their competencies) should be measured routinely. Follow-up studies should look at local outbreaks (last outbreak, number of cases, etc.) and demographics (population size, age structure, etc.), and make use of the full article, as opposed to just headlines to explore commonly given messages by news organizations for accuracy and tailor guidelines accordingly. This more in-depth analysis could also uncover whether public health officials were used as a source for information included in the article and their participation in the conversation with the public.

## 4. Materials and Methods

### 4.1. Surveys

The purpose of the interviews was to gather information on how mosquito surveillance and control is conducted in the CONUS in response to WNV. Among the areas of interest, we intended to evaluate how vector control agencies and personnel perceive the risk of WNV and other vector-borne diseases within their jurisdictions and what surveillance and vector control methods they use to detect the presence of viruses and control vector mosquitoes. A total of 24 organizations were interviewed from May 2020 to June 2021 with “purposive sampling” [33,37]; 10 more organizations had not been interviewed by manuscript preparation; we tried to survey the greatest number of states, while avoiding the overrepresentation of any single state. However, data should be considered non-random. The full list of questions is provided with the supplemental material (File S1).

We used two questions from the survey for the purpose of this research. (1) “What are the vector-borne diseases of concern within your jurisdiction?” Additionally, (2) “What mosquito species drive vector-borne disease transmission within your jurisdiction?” The interviews were composed of open answer questions; however, the answers were transformed to dichotomous variables; for example, from question 2 we created a list of all the unique species mentioned across all the interviews. Later from the list of species, we created a matrix of agency vs. species placing a value of 0 (zero) if the agency did not mention the species and a 1 (one) if the species was mentioned.

The number of mentions of diseases and vectors were analyzed with a multinomial test [38]. Algebraically that can be represented as *Mentions of disease (or vector)* ~ **Multinomial** (*proportion (disease or vector)*, *Total number of Mentions*). We used Bayesian inference and “rjags” [39] to estimate the *proportion* parameter for each of the diseases and vectors. We used uninformative priors (i.e., *proportion* ~ **Dirichlet** (*α* = 1)).

### 4.2. Headline Analysis

We captured headlines from articles published from 1999 through 2020 using the “US Newsstream” database as provided by proquest.com (accessed date: 15 September 2021; ProQuest LLC, Ann Arbor, MI, USA). The full description of the search is provided in Figure S1. To reduce redundancy the pool of articles was limited to those with a full text. The most important search parameters were the presence of the terms “West Nile Virus” or “Zika Virus” in the full text or the headline of the news article; the WNV and ZIKV searches were conducted independently. The results were imported to R objects for further scrubbing and analysis. The scrubbing strategy is included with the supplemental material (Figure S1).

Regarding “duplication”, we considered that a news article was *not* duplicated but a reprint, if the article was published in several news outlets (e.g., syndication) or if the article was published on different dates in the same news outlet because this “republication” represented the public’s interest in the topic as determined by the outlet’s editor. For example, the news article titled “Rains propel West Nile Virus surge in San Bernardino County” by Jim Steinberg [40] was published in “The Sun” on 7 October 2015 and 15 December 2015, and in the “Inland Valley Daily Bulletin” on 20 December 2015. We considered this situation to be three publications of a WNV article.

The frequency of news publications that mentioned WNV and ZIKV was evaluated by frequency overtime; their corresponding headlines using a word cloud. A word cloud (tag cloud or wordle) is a visual representation of terms and their relative importance. The terms are usually single words, in our case we joined the words “West”, “Nile”, and “Virus” when found together to form “wnv”. The same was true for “Zika” and “Virus” to form “zv”. We presented the frequency of the terms using different font sizes.

### 4.3. Google Trends Analysis

The data presented at trends.google.com (accessed date: 15 September 2021) “is an unbiased sample of (...) Google search data”, categorized and aggregated around a topic [41]. The idea behind this web tool is to measure interest across search terms. We searched for “West Nile Virus”, “Zika Virus”, and to contextualize the results we also added “Malaria” to the search. We removed data that was not part of the CONUS. Google provided monthly data starting in 2004. This data set did not include 1999–2003, the first five years of the WNV invasion of the CONUS. Google does not provide the actual number of searches, instead it creates a relative value where the month with the highest number of searches is given a value of 100%. Google searches represent an active event that is executed by an interested party, while a newspaper publication indicates the public’s interest as evaluated by individual news organizations.

## Figures and Tables

**Table 1:** Total number of counties in CONUS, and their corresponding states, with at least one neuroinvasive WNV report by date.

Years	Counties/States; First Report	Counties/States; Last Report
1999–2004	Affected counties: 1223 ^¶^; AL, AZ, AR, CA, CO, CT, DE, DC, FL, GA, ID, IL, IN, IA, KS, KY, LA, MD, MA, MI, MN, MS, MO, MT, NE, NV, NH, NJ, NM, NY, NC, ND, OH, OK, PA, RI, SC, SD, TN, TX, UT, VA, WV, WI, WY.	Affect. CTYs: 246; AL, AR, CA, CO, CT, FL, GA, IL, IN, IA, KS, KY, LA, MD, MI, MN, MS, MO, MT, NE, NH, NM, NY, NC, ND, OH, OK, PA, RI, SD, TN, TX, VA, WV, WI, WY
2005–2009	Affect. CTYs: 347; AL, AZ, AR, CA, CO, FL, GA, ID, IL, IN, IA, KS, KY, LA, MI, MN, MS, MO, MT, NE, NV, NM, NY, NC, ND, OH, OK, OR, SC, SD, TN, TX, UT, VA, WA, WV, WI.	Affect. CTYs: 211; AL, AR, CA, CO, FL, GA, ID, IL, IN, IA, KS, KY, LA, MN, MS, MO, MT, NE, NV, NM, NY, NC, ND, OH, OK, OR, PA, SD, TN, TX, UT, VA, WA, WV, WI.
2010–2014	Affect. CTYs: 257; AL, AZ, AR, CA, CO, FL, GA, ID, IL, IN, IA, KS, KY, LA, ME, MD, MA, MI, MN, MS, MO, MT, NE, NH, NJ, NM, NY, NC, ND, OH, OK, OR, PA, RI, SC, SD, TN, TX, VT, VA, WA, WV, WI, WY, PR.	Affect. CTYs: 373; AL, AZ, AR, CA, CO, CT, DE, FL, GA, ID, IL, IN, IA, KS, KY, LA, MD, MI, MN, MS, MO, MT, NE, NH, NJ, NM, NY, NC, ND, OH, OK, OR, PA, RI, SC, SD, TN, TX, UT, VT, VA, WA, WV, WI, WY, PR.
2014–2019	Affect. CTYs: 209; AL, AK, AR, CA, CO, CT, FL, GA, IL, IN, IA, KS, KY, LA, MD, MA, MI, MN, MS, MO, MT, NE, NJ, NM, NY, NC, ND, OH, OK, OR, PA, SC, SD, TN, TX, UT, VT, VA, WA, WV, WI.	Affect. CTYs: 1206; AL, AK, AZ, AR, CA, CO, CT, DE, DC, FL, GA, ID, IL, IN, IA, KS, KY, LA, ME, MD, MA, MI, MN, MS, MO, MT, NE, NV, NJ, NM, NY, NC, ND, OH, OK, OR, PA, RI, SC, SD, TN, TX, UT, VT, VA, WA, WV, WI, WY

Data source: ArboNET; WNV: West Nile Virus; CONUS: Continuous Unites States; ^¶^ In the 1999–2004 period, out of all counties with at least one neuroinvasive WNV case, 1223 counties had their first report (gray column), while only 246 counties had their last report in this period (white column).

**Table 2:** Total number of counties in CONUS, and their corresponding states, with at least one locally acquired ZIKV report by date.

Years	Counties/States; First Report	Counties/States; Last Report
2016	Affected counties: 7/38 ^¶^; CA, DC, FL *, GA, IL, LA, MD, MA, MN, MS, MO, NV, NH, NJ, NY, NC, OH, OR, PA, SC, TX *, VA	Affect. CTYs: 7/34; CA, DC, FL *, GA, IL, LA, MD, MA, MN, MS, MO, NV, NH, NJ, NY, NC, OH, OR, PA, SC, TX *, VA
2017	Affect. CTYs: 3/5; CA, FL *, MA, TX *	Affect. CTYs: 3/8 ^¶^; CA, FL *, MA, NY, TX *
2018	-	Affect. CTYs: 1/1 ^¶^; FL *

Data source: ArboNET; ArboNET; WNV: West Nile Virus; CONUS: Continuous Unites States; ^¶^ The first number represents the number of counties with mosquito transmission, the second number is the total number of cases (the gray column represents counties and states with a first report for the selected year); sexually transmitted cases = first number–second number; * States with mosquito transmission.

**Table 3:** Number of news articles related to WNV per newspaper by time, the total number (Sum) articles found and the percentage (%) they represent in the total dataset; complete dataset in Table S1; a map for “places of publication” can be found at https://www.newspapers.com/map/ accessed on 15 September 2021.

Newspaper/Pub. Year	1999	00–04	05–09	10–14	15–19	20	Sum	%
*Daily Herald*	-	430	275	202	188	22	1117	3%
*Newsday*	51	593	153	156	126	3	1082	3%
*Boston Globe*	3	441	185	98	100	11	838	2%
*Chicago Tribune*	9	398	199	111	52	9	778	2%
*New York Times*	52	492	111	51	33	7	746	2%
*The Washington Post*	11	466	104	85	56	17	739	2%
*The Journal News*	68	444	101	78	34	2	727	2%
*The Sun*	6	323	189	113	86	-	717	2%
*St. Louis Post-Dispatch*	7	345	104	122	18	2	598	2%
*Oakland Tribune*	-	50	318	149	59	-	576	2%
*New York Daily News*	40	389	42	50	3	1	525	1%
*Fort Collins Coloradoan*	-	223	119	93	80	8	523	1%
*Orlando Sentinel*	15	394	56	28	19	3	515	1%
*News-Star*	-	228	118	84	37	1	468	1%
*Hartford Courant*	15	310	50	47	39	2	463	1%
Others	202	11,983	6241	5045	3091	265	26,827	72%
						Total	37,239	100%

**Table 4:** Number of news articles related to ZIKV per newspaper by time, the total number (Sum) articles found and the percentage (%) they represent in the total dataset; complete dataset in Table S1; a map for “places of publication” can be found at “https://www.newspapers.com/map/ accessed on 15 September 2021”.

Newspaper/Pub. Year	2007	13	14	15	16	17	18	19	20	Sum	%
*Wall Street Journal*	-	-	-	3	521	44	19	15	27	629	5
*New York Times*	1	-	-	2	376	52	18	14	43	506	4
*The Washington Post*	-	-	-	1	348	42	16	14	45	466	4
*The Examiner*	-	-	-	-	333	19	10	6	5	373	3
*S. Florida Sun-Sentinel*	-	-	-	-	285	36	8	11	4	344	3
*Orlando Sentinel*	-	-	-	-	262	30	10	3	4	309	2
*Boston Globe*	-	-	-	-	216	36	9	12	27	300	2
*USA Today*	-	-	-	1	216	16	4	11	39	287	2
*Dayton Daily News*	-	-	-	1	219	32	1	-	-	253	2
*Chicago Tribune*	-	1	1	3	202	9	8	7	5	236	2
*Tampa Bay Times*	-	-	-	-	157	27	7	1	9	201	2
*Los Angeles Times*	-	-	-	-	151	14	10	11	8	194	1
*Newsday*	-	-	-	1	126	32	7	1	9	176	1
*The News Press*	-	-	-	-	138	20	6	1	9	174	1
*St. Louis Post-Dispatch*	-	-	-	1	142	13	4	2	2	164	1
Others	4	-	1	16	6799	877	297	125	246	8365	64
									Total	12,977	100

## Data Availability

We provide the interviews’ coded results as a Supplemental File S1. Headline copyright is a gray area of the law; therefore, we do not share them.

## Supplementary Materials

The following are available online at https://www.mdpi.com/article/10.3390/pathogens10121562/s1; Figure S1: Headline scrubbing process; File S1: Survey of Vector Control Agencies; Table S1: Articles in the final dataset by newspaper and year.
