# Supplementary material for: Perceived Risk of Mosquito-Borne Arboviruses in the Continental United States
Source: Pathogens. 2021 Nov 30;10(12):1562. doi: 10.3390/pathogens10121562 (PMC8706029; doi:10.3390/pathogens10121562)
Supplement: Supplementary file 1 [file pathogens-10-01562-s001.zip › file_s1.pdf]

Perceived risk of mosquito-borne arboviruses in the continental United States.

Saul Lozano[1,*], Jonathan Day[2] , Lilyana Ortega[3],Maggie Silver[1] and Roxanne Connelly[1]

File S1: Survey of Vector Control Agencies; The following is the list of open-ended question made to vector control agencies.

Q1. What are the vector-borne diseases of concern within your jurisdiction?

Q2. What mosquito species drive vector-borne disease transmission within your jurisdiction?

Q3. What wild avian species amplify WNV within your jurisdiction?

Q4. Where is WNV amplified within your jurisdiction?

Q5. When is WNV amplified within your jurisdiction?

Q6. What surveillance methods do you use to detect the presence of arboviruses within your jurisdiction?

Q7. How do you document changes in viral and mosquito abundance over time?

Q8. What proactive and reactive vector control strategies are used within your jurisdiction to reduce vector populations?

Q9. How do you evaluate vector control efforts during mosquito outbreaks?

Q10. Have you had success in reducing vector-borne disease transmission during an outbreak? How were the successes documented and evaluated?
