# Supplementary figures and images for: Perceived Risk of Mosquito-Borne Arboviruses in the Continental United States
Source: Pathogens. 2021 Nov 30;10(12):1562. doi: 10.3390/pathogens10121562 (PMC8706029; doi:10.3390/pathogens10121562)

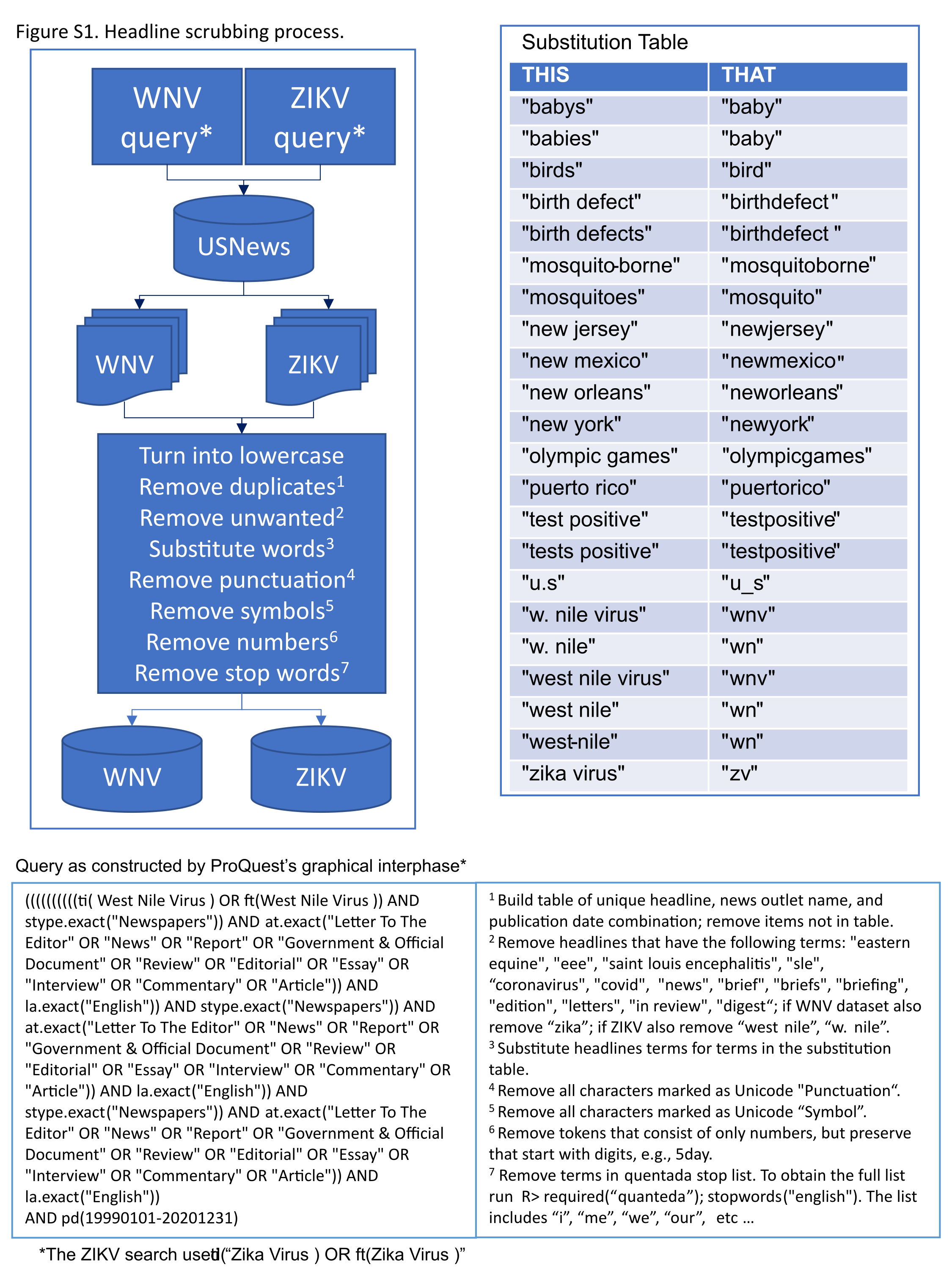

Supplement: Supplementary file 1 [file pathogens-10-01562-s001.zip › figure_s1.png]
